# The Protective Effects of Lipid-Lowering Agents on Cardiovascular Disease and Mortality in Maintenance Dialysis Patients: Propensity Score Analysis of a Population-Based Cohort Study

**DOI:** 10.3389/fphar.2021.804000

**Published:** 2022-01-28

**Authors:** Ming-Hsien Tsai, Mingchih Chen, Yen-Chun Huang, Hung-Hsiang Liou, Yu-Wei Fang

**Affiliations:** ^1^ Division of Nephrology, Department of Internal Medicine, Shin-Kong Wu Ho-Su Memorial Hospital, Taipei City, Taiwan; ^2^ Department of Medicine, School of Medicine, Fu Jen Catholic University, New Taipei City, Taiwan; ^3^ Graduate Institute of Business Administration, College of Management, Fu Jen Catholic University, New Taipei City, Taiwan; ^4^ AI Development Center, Fu Jen Catholic University, New Taipei City, Taiwan; ^5^ Division of Nephrology, Department of Internal Medicine, Hsin-Jen Hospital, New Taipei City, Taiwan

**Keywords:** dialysis, National Health Insurance Research Database, lipid-lowering agents, mortality, major adverse cardiovascular events

## Abstract

Lipid-lowering agents display limited benefits on cardiovascular diseases and mortality in patients undergoing dialysis. Therefore, they are not routinely recommended for dialysis patients. The aim of this study was to assess the effects of lipid-lowering agents on clinical outcomes in dialysis patients on the basis of real-world evidence. This research used Taiwan’s National Health Insurance Research Database to identify dialysis patients from January 2009 to December 2015; patients were then categorized into a case group treated with lipid-lowering agents (*n* = 3,933) and a control group without lipid-lowering agents (*n* = 24,267). Patients were matched by age, sex, and comorbidities in a 1:1 ratio. This study used the Cox regression model to estimate the hazard ratios (HRs) for mortality and major adverse cardiovascular events (MACEs) for events recorded until December 2017. During a mean follow-up period of approximately 3.1 years, 1726 [43.9%, incidence 0.123/person-year (PY)] deaths and 598 (15.2%, incidence 0.047/PY) MACEs occurred in the case group and 2031 (51.6%, incidence 0.153/PY) deaths and 649 (16.5% incidence 0.055/PY) MACEs occurred in the control group. In the multivariable analysis of the Cox regression model, lipid-lowering agent users showed a significantly lower risk of death [HR: 0.75; 95% confidence interval (CI): 0.70–0.80] and MACEs (HR: 0.88; 95% CI: 0.78–0.98) than lipid-lowering agent non-users. Moreover, the survival benefit of lipid-lowering agents was significant across most subgroups. Dialysis patients treated with lipid-lowering agents display a 25 and 12% reduction in their risk of mortality and MACEs, respectively. Therefore, lipid-lowering agents might be considered when treating dialysis patients with hyperlipidemia.

## Introduction

Patients with chronic kidney disease (CKD) are at a high risk of morbidity and mortality owing to cardiovascular disease (CVD) (Jankowski et al., 2021); the risk of CVD starts from an early CKD stage and increases along with a decline in the estimated glomerular filtration rate (Yu et al., 2021). While entering into dialysis-dependent end-stage renal disease (ESRD), the risk of CVD significantly increases compared with that in the general population, accounting for >50% of the mortality (Chronic Kidney Disease Prognosis Consortium et al., 2010; Sharma and Sarnak, 2017; Johansen et al., 2021).

Dyslipidemia is a traditional risk factor contributing to CVD, and its correction has been considered the mainstay of treatment in high-risk groups (Lee et al., 2017; Hedayatnia et al., 2020). The first large-scale study investigating the effects of lipid-lowering agents was the Scandinavian Simvastatin Survival Study (4S), which demonstrated that this statin reduced cardiovascular events and mortality in patients with coronary artery disease (Pedersen et al., 1994). Recently, the benefit of statin therapy in CVD is well studied, and it is recommended for the primary prevention of CVD in those at a high risk of atherosclerosis (Ziaeian and Fonarow, 2017; Arnett et al., 2019; Byrne et al., 2019). Furthermore, some studies have suggested that achieving a very low level of low-density lipoprotein cholesterol (LDL-C) (<30 mg/dl) using high-intensity statin was safe and beneficial for the very high–risk population (Nicholls et al., 2016; Giugliano et al., 2017a; Giugliano et al., 2017b). However, the mechanisms and impacts of dyslipidemia remain unclear in patients with ESRD undergoing dialysis. The most widely accepted hypothesis is the carbamylation of LDL-C in the uremia stage (Kraus and Kraus, 2001; Ok et al., 2005). Carbamylated LDL has been shown to be a potent atherogenic factor *in vitro* and *in vivo.* Through endothelial cell damage, the degree of vascular smooth muscle cell proliferation and endothelial cell apoptosis was proportional to the degree of LDL carbamylation and subsequently induced accelerated atherosclerosis (Ok et al., 2005; Apostolov et al., 2010).

Because dyslipidemia is prevalent in patients with ESRD undergoing dialysis (Qunibi, 2015), two large randomized controlled trials have been conducted to investigate its correction by using lipid-lowering agents in merely dialysis patients. The Die Deutsche Diabetes Dialyze Studie (4D study) and A Study to Evaluate the Use of Rosuvastatin in Subjects on Regular Hemodialysis: An Assessment of Survival and Cardiovascular Events (AURORA) showed no benefit of statin use in dialysis patients (Wanner et al., 2005; Fellström et al., 2009). Moreover, the Study of Heart and Renal Protection (SHARP) including both non–dialysis-dependent CKD and dialysis patients showed that statin therapy did not significantly reduce the major adverse cardiovascular events (MACEs) in the subgroup of dialysis patients (hemodialysis and peritoneal dialysis) (Baigent et al., 2011). Therefore, lipid-lowering agents have not been routinely recommended for dialysis patients with hyperlipidemia owing to the aforementioned results in the current guideline (Mach et al., 2020).

Conversely, some previous observational studies have reported that lipid-lowering therapy is associated with reduced mortality in dialysis patients (Seliger et al., 2002; Mason et al., 2005; Jung et al., 2020). A recent study using a population-based nationwide dataset in Korea showed that statins combined with ezetimibe significantly lowered the all-cause mortality in adult patients undergoing maintenance hemodialysis (Jung et al., 2020), and the finding of which suggested that the application of lipid-lowering agents in the dialysis population might be re-evaluated. Moreover, hypertriglyceridemia is commonly observed in dialysis patients, but the therapeutic benefit of using the fenofibrate to lower triglyceride levels is unclear in this group (Hung et al., 2009; Mikolasevic et al., 2017). However, some studies had reported that the use of fenofibrate can provide cardiovascular and mortality benefits in non-dialysis CKD patients (Ting et al., 2012; Yen et al., 2021). Accordingly, we conducted this study to investigate whether the risk of MACEs and all-cause mortality could be reduced by lipid-lowering agents (statin and fenofibrate) in dialysis patients, including hemodialysis and peritoneal dialysis, on the basis of a real-world database.

## Materials and Methods

### Data Sources and Research Samples

In this retrospective study, the source of information was the National Health Insurance Research Database (NHIRD). The NHIRD was established in 1996, including coverage for nearly 99% of beneficiaries who were certified citizens in Taiwan since 1998, and it provides diverse medical information, with inpatient and outpatient demographics, clinical records, diagnosis, procedure codes, and medical expenses (Hsieh et al., 2019). The diagnosis code was based on the International Classification of Diseases (9th and 10th) Clinical Modification (ICD-9-CM; ICD-10-CM) codes. The benefits of using NHIRD include the access to long-term follow-up data.

This study was performed in accordance with the principles of the Declaration of Helsinki and was approved by the Institutional Review Board of Fu Jen Catholic University (IRB approval number: No. C104016). All claim records were anonymized before analysis, and the requirement of written informed consent was waived by the Institutional Review Board of Fu Jen Catholic University.

### Study Population and Exclusion Criteria

**FIGURE 1:**
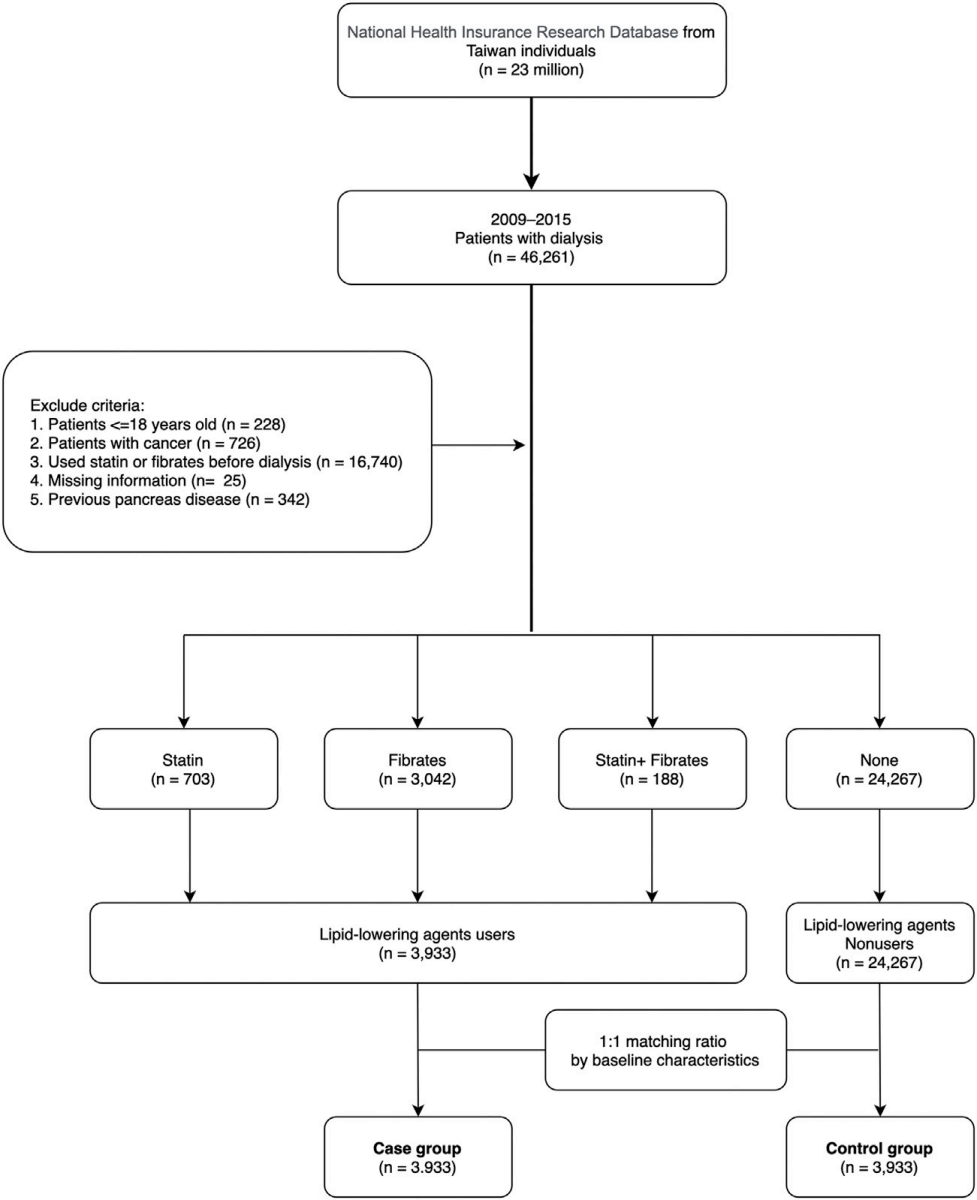
Schematic of patient enrollment in the study.

The data for this study were collected between 1 January 2009 and 31 December 2015. Patients undergoing peritoneal dialysis or hemodialysis for more than 3 months were included (*n* = 46,261). The codes of dialysis procedures are shown in Supplementary Table S1. Exclusion criteria were age less than 18 years (*n* = 228), missing information (*n* = 25), cancer diagnosis (*n* = 726), use of statin or fibrates before entering the dialysis-dependent CKD stage (*n* = 16,740), and having multiple pancreatic diseases (acute pancreatitis, complications pancreas transplantation, pancreas damage of head, chronic inflammatory pancreatitis, and defect of pancreas, *n* = 342) before the index date. A total of 28,200 patients were categorized into case and control groups, and cases using lipid-lowering agents (including statin and fibrate for 2 months) (*n* = 3,933) were matched with controls (*n* = 24,267) who did not use lipid-lowering agents within 6 months after entering the dialysis-dependent CKD stage. To reduce bias in our research, we used a 1:1 ratio propensity score matching to the baseline information including sex, age, baseline comorbidities, Charlson comorbidity index score (CCIS) (Charlson et al., 1987), and hospital area (Figure 1).

Baseline comorbidities, namely, hypertension, diabetes mellitus, hyperlipidemia, ischemic heart disease, chronic heart failure, stroke, peripheral artery occlusion disease, chronic obstructive pulmonary disease, liver disease, and biliary stone, were defined as diseases when they had at least three outpatient diagnoses or one inpatient diagnosis within 1 year before the index date (Supplementary Table S1). The usage of other drugs, namely, angiotensin-converting enzyme inhibitors/angiotensin receptor blockers (ACEI/ARB), calcium channel blockers (CCB), beta-blockers, anticoagulants, digoxin, dipeptidyl peptidase-4 inhibitor (DDP4i), insulin, nonsteroidal anti-inflammatory drugs (NSAIDs), uric acid–lowering agents, and benzodiazepines was considered when the patients used the drug for at least 2 months within 6 months before the index date. The prescription of implanted cardioverter defibrillators (ICDs) was also collected before the index date (Supplementary Table S2).

### Clinical Outcomes

The index date for users was the date after using lipid-lowering agents for 2 months and that for non-users was the date after entering dialysis for 6 months. Patients were followed up until the occurrence of clinical events including all-cause death, ischemic stroke, and MACEs (the composite cardiovascular events: myocardial infarction, cerebrovascular disease, heart failure, and arrhythmia), or the end of the research (2017-12-31), whichever occurred first. The date of death was collected by linking to the database of the National Register of Deaths. Stroke and MACEs were mainly diagnosed according to the ICD code (Supplementary Table S1).

### Statistical Analysis

Continuous data are expressed as mean ± standard deviation and categorical data as proportions (%). Categorical variables were analyzed using chi-squared tests. Continuous variables were analyzed using paired *t*-tests to validate demographic characteristics, including sex and age, and baseline comorbidities between case and control groups. The probability of propensity score was defined by covariates and estimated by using a logistic or probit regression model. There are two important points in propensity matching. First, the researchers have to make sure the matching ratio between the explored and unexplored groups. Second, researchers must determine which baseline variables were the confounding factors and eliminate them (Austin, 2008; Austin and Cafri, 2020). This research used propensity score matching with a 1:1 ratio and used baseline variables to eliminate the discrepancy between lipid-lowering agent users and non-users (Austin, 2011). An intention-to-treatment basis was adopted according to the patients’ initial lipid-lowering agent use status without consideration of the subsequent regimen change.

Event-free survival curves were created using the Kaplan–Meier method and tested with the log-rank test. Moreover, the Cox proportional hazard model was adopted to estimate the hazard ratio (HR) with 95% confidence intervals (CI) of clinical outcomes as a function of lipid-lowering agent use. The assumption of proportionality was not violated by testing for an interaction between time and the variables. We also performed a subgroup analysis stratified by comorbidities. All statistical analyses and figures were created using 9.4 version of SAS (SAS Institute, Cary, NC, United States) software. For all tests, two-tailed *p*-values of <0.05 were considered statistically significant.

## Results

### Patient Characteristics

We enrolled 28,200 dialysis patients in the present study (Figure 1). Among them, 3,933 patients (13.9%) used lipid-lowering agents for at least 2 months after entering dialysis. The mean age of lipid-lowering agent users (case group) was 58.8 years, of whom 44.9% were men, 9.4% had hypertension, 43.7% had diabetes mellitus, 33.1% had coronary artery disease, and 19.2% had a stroke history. Moreover, 41.2% of patients were from northern Taiwan and 33.2% were from southern Taiwan (Table 1). Before matching, lipid-lowering agent users had lesser comorbidities than lipid-lowering agent non-users. However, the baseline demographic data and comorbidities between lipid-lowering agent users and non-users showed no significant difference after matching. For baseline medical prescriptions after matching, lipid-lowering agent users had a higher prescription rate in ACEI/ARB, CCB, beta-blockers, anticoagulants, DPP4i, insulin, and uric acid–lowering agents than in lipid-lowering agent non-users after matching, as shown in Table 1.

**TABLE 1:** Demographic and clinical characteristics of dialysis patients.

Variable	Lipid-lowering agents				
	Before matching			After matching	
	Users (*n* = 3,933)	Non-users (*n* = 24,267)	*p* Value	Non-users (*n* = 3,933)	*p* Value
Gender					
Male	1,765 (44.9)	12,612 (52.0)	<0.001	1,760 (44.8)	0.909
Female	2,168 (55.1)	11,655 (48.0)		2,173 (55.3)	
Age (years), mean (SD)	58.8 (13.8)	63.3 (15.1)	<0.001	59.0 (14.5)	0.512
Comorbidities					
Hypertension	371 (9.4)	2,543 (10.5)	0.045	422 (10.7)	0.056
Diabetes mellitus	1,717 (43.7)	9,801 (40.4)	<0.001	1,686 (42.9)	0.480
Hyperlipidemia	1,662 (42.3)	4,805 (19.8)	<0.001	1,630 (41.4)	0.464
CAD	1,302 (33.1)	7,456 (30.7)	0.002	1,266 (32.2)	0.386
CHF	1,356 (34.5)	8,863 (36.5)	0.013	1,351 (34.4)	0.905
Stroke	756 (19.2)	5,699 (23.5)	<0.001	799 (20.3)	0.223
PVD	526 (13.4)	3,932 (16.2)	<0.001	587 (14.9)	0.048
COPD	729 (18.5)	6,062 (25.0)	<0.001	713 (18.1)	0.641
Liver disease	924 (23.5)	7,886 (32.5)	<0.001	845 (21.5)	0.032
Biliary stone	213 (5.42)	1,887 (7.8)	<0.001	232 (5.9)	0.353
CCIS, mean (SD)	3.5 (2.7)	4.0 (2.9)	<0.001	3.5 (2.9)	0.876
Hospital area					
Central	916 (23.3)	6,208 (25.6)	0.007	979 (24.9)	0.355
Northern	1,622 (41.2)	9,892 (40.8)		1,605 (40.8)	
Southern	1,306 (33.2)	7,576 (31.2)		1,256 (31.9)	
Eastern	89 (2.3)	591 (2.4)		93 (2.4)	
Prescription					
ACEI/ARB	1,107 (28.2)	5,352 (22.1)	<0.001	989 (25.2)	0.002
CCB	2,025 (51.5)	11,083 (45.7)	<0.001	1,904 (48.4)	0.006
Beta-blocker	1,672 (42.5)	8,180 (33.7)	<0.001	1,477 (37.6)	<0.001
Anticoagulants	1,870 (47.6)	10,343 (42.6)	<0.001	1,658 (42.2)	<0.001
Digoxin	62 (1.6)	478 (2.0)	0.095	73 (1.9)	0.339
ICDs	67 (1.7)	386 (1.6)	<0.001	70 (1.8)	0.796
DPP4i	440 (11.2)	1,415 (5.8)	<0.001	280 (7.1)	<0.001
Insulin	977 (24.8)	4,799 (19.8)	<0.001	851 (21.6)	<0.001
NSAID	1,648 (41.9)	10,597 (43.7)	0.038	1,656 (42.1)	0.855
UA-lowering agents	478 (12.2)	1,953 (8.1)	<0.001	367 (9.3)	<0.001
Benzodiazepines	1,817 (46.2)	11,498 (47.4)	0.168	1,776 (45.2)	0.353

Abbreviation: SD, standard deviation; CAD, coronary artery disease; CHF, congestive heart failure; PVD, peripheral vascular disease; COPD, chronic obstructive pulmonary disease; CCIS, Charlson Comorbidity Index Score; ACEI/ARB, angiotensin-converting enzyme inhibitors/angiotensin receptor blockers; CCB, calcium channel blockers; ICDs, implanted cardioverter defibrillators; DPP4i, dipeptidyl peptidase-4, inhibitor; NSAID, non-steroidal anti-inflammatory drug; UA-lowering agents, uric acid–lowering agents.

In the group of lipid-lowering agents users, the numbers of patients who did not use lipid-lowering agents after the index day were 157 (4%) within 1 year and 297 (7.5%) within 2 years. However, the non-users of lipid-lowering agents were kept free from the lipid-lowering agents for 1 and 2 years after the index day.

### Benefit of Using Lipid-Lowering Agents in Dialysis Patients

**FIGURE 2:**
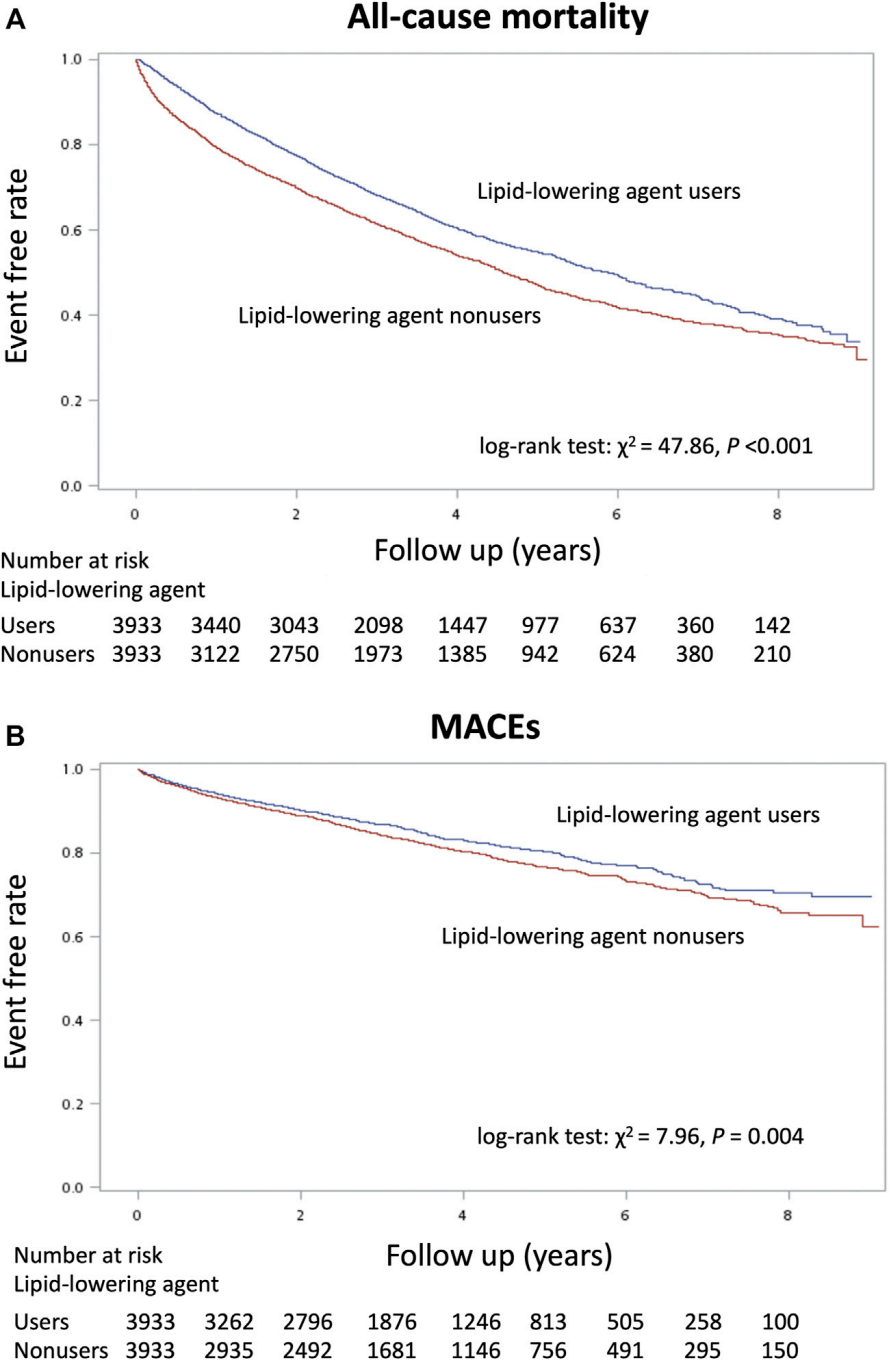
Kaplan–Meier cumulative event-free plots of **(A)** mortality and **(B)** major adverse cardiovascular events (MACEs) in the study population according to whether the lipid-lowering agents were used or not.

The total follow-up summation is 86,627 person-year (PY) during the study period (Table 2). A total of 16,866 patients (59.8%) died, 4,512 patients (16%) had a new-onset MACE, and 954 patients (3.4%) experienced the ischemic stroke. Before matching, the lipid-lowering agent users had a better incidence rate than non-users in mortality (0.123/PY vs 0.208/PY) and MACEs (0.47/PY vs 0.60/PY) but not in ischemic stroke (0.01 vs 0.01%). Moreover, the Kaplan–Meier event-free curves for all-cause mortality (Figure 2A) and MACEs (Figure 2B) among lipid-lowering agents users compared with non-users were both significant (*p* < 0.05) after matching. This finding indicated that the use of lipid-lowering agents was associated with a lower risk of mortality and MACEs.

**TABLE 2:** Risk of clinical outcomes in patients with dialysis comparing lipid-lowering agents users vs. non-users.

Clinical outcome	Before matching					After matching			
	Users (*n* = 3,933)		Non-users (*n* = 24,267)		Users vs Non-users	Non-users (*n* = 3,933)		Users vs non-users	
								Model 1	Model 2
	Events	IR	Events	IR	HR (95%CI)	Events	IR	HR (95%CI)	HR (95%CI)
All-cause mortality	1726	12.30	15,140	20.8	0.60** (0.57–0.63)	2031	15.3	0.80** (0.75–0.85)	0.75** (0.70–0.80)
MACEs	598	4.70	3,914	6.00	0.79* (0.72–0.86)	649	5.50	0.85* (0.76–0.95)	0.88* (0.78–0.98)
Ischemic stroke	137	1.00	817	1.10	0.87 (0.72–1.04)	138	1.10	0.93 (0.73–1.18)	0.87 (0.69–1.11)

Model 1 is crude analysis after matching. Model 2 is further adjusted by medical prescriptions, including angiotensin-converting enzyme inhibitors/angiotensin receptor blockers, calcium channel blockers, beta-blockers, anticoagulants, digoxin, implanted cardioverter defibrillators, dipeptidyl peptidase-4, inhibitor, insulin, non-steroidal anti-inflammatory drugs, uric acid–lowering agents, and benzodiazepines.

Abbreviation: IR, incidence rate (in every 100 person-years); HR, hazard ratio; CI, confidence interval; aHR, adjusted hazard ratio; MACEs, major adverse cardiovascular events.

*p < 0.001. **p < 0.05.

As shown in Table 2, we found that treatment with lipid-lowering agents in dialysis patients significantly decreased their risk of mortality (HR, 0.60; 95% CI, 0.57–0.63) and MACEs (HR, 0.79; 95% CI, 0.72–0.86) before matching, and the beneficial effects of lipid-lowering agents were maintained after propensity score matching [HR: 0.80 (0.75–0.85); 0.85 (0.76–0.95), respectively]. After further adjusting the medical prescriptions, such significance was also kept [HR: 0.75 (0.70–0.80) for mortality; 0.88 (0.78–0.98) for MACEs]. The risk of ischemic stroke event had no significant difference between the matching groups [HR: 0.93 (0.73–1.18) in the crude model; 0.87 (0.69–1.11) in the multivariable adjusting model].

In Table 3, it shows the events of composites of MACEs between lipid-lowering agents users and non-users during the observation period, of which myocardial infarction had a significant difference between groups (3.5 vs 2.4%, *p* = 0.003).

**TABLE 3:** Components of MACEs between patients with dialysis comparing lipid-lowering agents users vs. non-users.

Components of MACEs	After matching		
	Users (*n* = 3,933)	Non-users (*n* = 3,933)	*p* value
Myocardial infarction	138 (3.5)	94 (2.4)	0.003
Heart failure	319 (8.1)	346 (8.8)	0.273
Cerebrovascular disease	236 (6)	253 (6.4)	0.427
Cardiac arrhythmia	184 (4.7)	211 (5.4)	0.163

Abbreviation: MACEs, major adverse cardiac events.

### Subgroup Analysis

**FIGURE 3:**
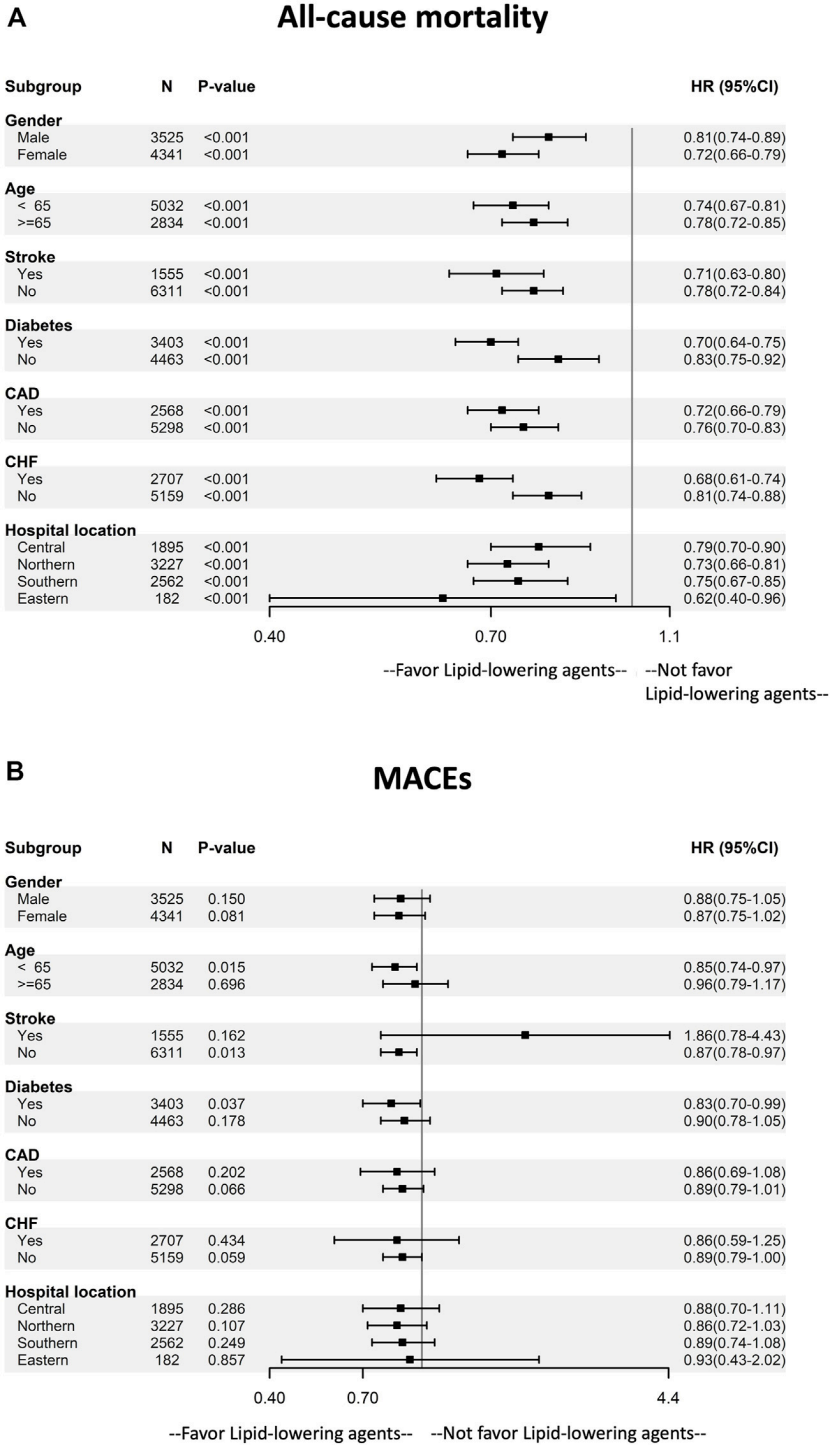
Subgroup analysis of the effect of lipid-lowering agents use on **(A)** mortality and **(B)** MACEs by baseline characteristics. The multivariable adjusting model was the same as model 2 in Table 2. Abbreviation: CAD, coronary artery disease; CHF, congestive heart disease; MACEs, major adverse cardiovascular events.

We conducted a series of stratified analyses to test the reliability of our results (Figure 3). The decreased HRs of mortality among dialysis patients in favor of lipid-lowering agents were consistent across all patient subgroups. Patients of young age, those with diabetes, and those without a history of stroke and congestive heart failure showed a significantly lower risk of MACEs.

## Discussion

We have provided real-world evidence on the effects of lipid-lowering agents in reducing cardiovascular events and mortality in dialysis patients using an NHIRD dataset. This finding is crucial to disclose that dyslipidemia correction still plays a role in the improvement of CVD and mortality in dialysis patients, which belong to the high-risk CVD group. Our study extends the current knowledge about the use of lipid-lowering agents in the dialysis population who were eligible to use lipid-lowering agents which may be associated with the reduced mortality and MACEs, and the risk reduction of MACEs may be attributed to the prevention of myocardial infarction. Therefore, we suggested that lipid-lowering agents (stain or fenofibrate) may be applied to dialysis patients with dyslipidemia.

There has been a longstanding debate regarding the use of lipid-lowering agents in chronic dialysis patients. However, this question became unequivocal since the outcomes of the 4D, AURORA, and SHARP trials revealed negative results in the dialysis population without overt CAD (Wanner et al., 2005; Fellström et al., 2009; Baigent et al., 2011). In the 4D study, diabetes patients undergoing chronic hemodialysis were randomly assigned to receive a statin (atorvastatin) or a placebo. In the statin-treated group, LDL cholesterol levels significantly reduced from baseline since the first 4 weeks and were maintained for 5 years. This reduction in LDL cholesterol did not offer benefit in reducing MACEs compared with placebo, suggesting that it was too late to use statin in the dialysis population (Wanner et al., 2005). Regarding the AURORA study conducted in hemodialysis patients with or without diabetes, similar results were noted between the statin (rosuvastatin) and placebo groups (Fellström et al., 2009). Moreover, the SHARP study showed that after an average follow-up period of 4.9 years, a significant reduction in major atherosclerotic events was observed in the recruited participants. However, this positive finding was not significant in the subgroup based on dialysis at enrollment (Baigent et al., 2011). Moreover, a meta-analysis combined these three studies and showed that statin use had a significant benefit on nonfatal atherosclerotic CV events [odds ratio (OR), 0.89, 95% CI, 0.8–0.99] but not on coronary events (OR, 0.97; 95% CI, 0.88–1.06), ischemic stroke (OR, 1.29, 95% CI, 0.72–2.31), and all-cause mortality (OR, 0.88; 95% CI, 0.74–1.04) (Green et al., 2013).

Because the effects of lipid-lowering agents in dialysis patients were not as expected for the general population in these large randomized controlled trials (RCTs), there are several possible explanations for these negative results. The first one is the complexity of CVD in patients undergoing dialysis. Apart from traditional risk factors, several types of pathogenesis contribute to atherosclerosis and the decline in heart function. The so-called non-traditional factors include mineral and bone metabolism disorder, oxidative stress, and anemia (Yao et al., 2004). Uremic toxin, p-cresol, and indoxyl sulfate have been linked with atherosclerosis and vascular calcification (Barreto et al., 2009; Opdebeeck et al., 2020). In addition, the increased level of fibroblast growth factor 23 as an indicator of renal function decline has shown its deleterious effects on cardiomyocytes (Gutiérrez et al., 2008). These factors render the association of a direct causal relationship between LDL cholesterol and atheroma less relevant (Mihaylova et al., 2012; Fulcher et al., 2015). In addition, there are aspects of CVD in ESRD, such as arrhythmia and heart failure, that affect non-atheromatous pathogenesis (Methven et al., 2017; Saran et al., 2017). Intervention in only one dimension of lipid control will not solve these issues as noted in these large RCT studies.

Despite the negative results from RCT trials, some studies found that ESRD groups undergoing dialysis could benefit from lipid-lowering agents. In the post hoc analysis of the 4D study, statin still lowered the risk of cardiovascular events when the LDL level was >145 mg/dl (März et al., 2011). This suggested that in dialysis patients with a severe degree of dyslipidemia, aggressive lipid-lowering therapy can also reduce cardiovascular mortality in a long-term observational period. Moreover, statin treatment in dialysis patients after acute myocardial infarction improves overall mortality (Chung et al., 2017). Statin treatment significantly decreases overall mortality in ESRD patients with acute myocardial infarction compared with the non-statin group. This is more prominent in the cardiac shock patient subgroup. These results are compatible with those from other studies, supporting a measurable benefit from statins in ESRD patients (Chung et al., 2017).

In our study using Taiwan’s NHIRD, chronic dialysis patients using lipid-lowering agents had a better outcome in MACEs, especially the composite of myocardial infarction, and all-cause mortality, suggesting that such agents may be helpful in this population. However, the reasons for the discordance between RCTs and the observational study findings about the use of lipid-lowering agents in dialysis patients are still undetermined. We proposed some possible explanations. In our previous single-center study cohort, we observed an inverted U-curve association between serum indoxyl sulfate levels and cardiovascular events in chronic hemodialysis patients (Tsai et al., 2021). The reported data indicate that although indoxyl sulfate, a recognized uremic toxin, may contribute to atherosclerosis and cardiovascular pathogenesis *in vivo* and *in vitro*, its higher levels led to a better cardiovascular outcome. In our observation, the underlying nutritional status may explain this reverse epidemiology (Zha and Qian, 2017; Hanna et al., 2020). Regarding the lipid-lowering issue, only well-nourished patients in chronic dialysis would be suitable to receive such agents. This may be a reasonable explanation for this finding in our population-based cohort study. Also, the other explanation is that RCTs have a good enteral validity but a relatively low external validity and generalization due to their highly selective population and tightly control setting. Indeed, in real-world practice, patients have higher heterogenicity than those in RCTs. Therefore, a discrepancy exists between RCTs and the studies using real-word data (Kim et al., 2018).

Our study had several strengths. First, we used data from a nationwide database, meaning that the study’s results can be generalized. Second, the sample size and observation time were adequate to obtain sufficient inferences. Despite its strengths, our study had several potential limitations. First, NHIRD, an original claim database for reimbursement, does not offer clinical information such as biochemical data, inflammatory burden, blood pressure, and body characteristics (weight, height, waist circumference, and body fat percentage) which might have an impact on the development of MACEs (Sardu et al., 2019; Sardu et al., 2021a; Sardu et al., 2021b). However, our study’s large number size with the method of propensity score matching can alleviate this bias. Second, we cannot determine whether the study patients showed regular drug compliance because exposure to lipid-lowering agents was based on prescription information only. Third, this was not a RCT; thus, the unbalanced baseline between the two groups was a major concern, which might induce a bias of confounding by indication (Kyriacou and Lewis, 2016). However, we used the propensity score matching to reduce this bias as the method of propensity score matching is well developed to balance the underlying difference between groups. Also, we further adjusted the medications and ICDs use, the devices could significantly ameliorate clinical cardiovascular outcomes (Sardu et al., 2017), and the significant results in our study were kept.

In conclusion, although lipid-lowering agents are not recommended for routine use in dialysis patients with hyperlipidemia according to the clinical guideline, our results demonstrated significant benefits of the use of lipid-lowering agents on clinical outcomes, including MACEs, and all-cause death, in such a population.

## Data Availability

The datasets presented in this article are not readily available because all claim records were anonymized before analysis. Thus, Fu Jen Catholic University Ethics Institutional Review Board was exempted from a full ethical review, and the requirement to obtain informed consent was waived. Requests to access the datasets should be directed to https://dep.mohw.gov.tw/dos/np-1714-113.html.
