# Supplementary material for: The Protective Effects of Lipid-Lowering Agents on Cardiovascular Disease and Mortality in Maintenance Dialysis Patients: Propensity Score Analysis of a Population-Based Cohort Study
Source: Front Pharmacol. 2022 Jan 28;12:804000. doi: 10.3389/fphar.2021.804000 (PMC8831748; doi:10.3389/fphar.2021.804000)
Supplement: Supplementary file 1 [file DataSheet2.PDF]

S2. Code for drugs

Drug type	ATC classification system codes	Drug name
ACEI/ARB	C09A, C09B, C09C, C09D	Captopril, Enalapril, Lisinopril, Perindopril, Ramipril, Quinapril, Benazepril, Cilazapril, Fosinopril, Imidapril, Losartan, Eprosartan, Valsartan, Irbesartan, Candesartan, Telmisartan, Olmesartan, Azilsartan
CCB	C08C, C08D, C09BB, C09DB, C09DX01, C09DX03 C09XA, C10BX	Amlopidine, Felodipine, Isradipine, Nicardipine, Nifedipine, Nimodipine, Nisoldipine, Nitrendipine, Lacidipine, Barnidipine, Lercanidipine, Cilnidipine, Benidipine, Verapamil, Diltiazem
Beta blocking agents	C07A, C07B, C07CA03, C07DA06	Alprenolol, Oxprenolol, Pindolol, Propranolol, Timolol, Sotalol, Nadolol, Carteolol, Bupranolol, Metoprolol, Atenolol, Acebutolol, Betaxolol, Bevantolol, Bisoprolol, Esmolol, Nebivolol, Labetalol, Carvedilol
Anticoagulants	B01AA, B01AB, B01AC, M01BA03, N02AB, N02BA01, N02BA51	Phenindione, Warfarin, Heparin, Dalteparin, Enoxaparin, Nadroparine, Tinzaparin, Clopidogrel, Ticlopidine, Acetylsalicylic Acid, Dipyridamole, Epoprostenol, Iloprost, Abciximab, Eptifibatide, Tirofiban, Treprostinil, Prasugrel, Cilostazol, Ticagrelor, Selexipag,
Digoxin	C01AA	Digitoxin, Digoxin, Deslanoside, Metildigoxin
DPP4	A10BH	Sitagliptin, Vildagliptin, Saxagliptin, Alogliptin, Linagliptin
Insulin	A10AB, A10AC, A10AD, A10AE	Insulin

NSAID	M01AB, M01AC, M01AE, M01AG, M01AH, M01AX01	Indomethacin, Sulindac, Tolmetin, Diclofenac, Alclofenac, Etodolac, Acemetacin, Ketorolac, Aceclofenac, Piroxicam, Tenoxicam, Meloxicam, Ibuprofen, Naproxen, Ketoprofen, Fenoprofen, Fenbufen, Flurbiprofen, Tiaprofenic Acid, Alminoprofen, Naproxen, Mefenamic Acid,,Tolfenamic Acid Flufenamic Acid, Meclofenamic Acid, Celecoxib, Rofecoxib, Etoricoxib, Nabumetone, Niflumic Acid
UA-lowering agents	M04AA01, M04AA03	Allopurinol, Febuxostat
Benzodiazepines	N05BA, N05CD	Diazepam, Chlordiazepoxide, Medazepam, Oxazepam, Clorazepate, Lorazepam, Bromazepam, Clobazam, Prazepam, Alprazolam, Nordazepam, Fludiazepam, Cloxazolam, Oxazolam, Flurazepam, Nitrazepam, Flunitrazepam, Estazolam, Triazolam, Lormetazepam, Temazepam, Midazolam, Brotizolam, Nimetazepam
**Code for Prescription**		
ICDs		Z95.810, V45.02

Abbreviation:

ACEI /ARB, Angiotensin-converting enzyme inhibitors /Angiotensin receptor blockers; CCB, Calcium channel blockers; DPP4, Dipeptidyl peptidase-4 inhibitor; NSAID, Non-Steroidal Anti-Inflammatory Drug; UA-lowering agents, Uric Acid-lowering agents; ICDs, implanted cardioverter defibrillators.
