# Supplementary material for: The Protective Effects of Lipid-Lowering Agents on Cardiovascular Disease and Mortality in Maintenance Dialysis Patients: Propensity Score Analysis of a Population-Based Cohort Study
Source: Front Pharmacol. 2022 Jan 28;12:804000. doi: 10.3389/fphar.2021.804000 (PMC8831748; doi:10.3389/fphar.2021.804000)
Supplement: Supplementary file 2 [file DataSheet1.PDF]

S1. Code for comorbidities and clinical outcomes

	ICD-9-CM	ICD-10-CM
Dialysis (Order Code)	Peritoneal dialysis: 58002C, 58009A, 58009B, 58010A, 58010B, 58011A, 58011AB, 58011B, 58011C, 58012A, 58012B, 58017B, 58017C, 58026C, 58028C<br>Hemodialysis:58001C, 58019C, 58020C, 58021C, 58022C, 58023C, 58024C, 58025C, 58027C, 58029C	
Comorbidities		
Hypertension	401 – 405	I10-14
Diabetes mellitus	250	E10-E14
Hyperlipidemia	272.0–272.4	E78
Coronary artery disease	410 – 414	I20-I25
Stroke	430–438	I60- I63, I65-I69, G45-G46
Liver disease	006.3,070,072.71,091.62,095.3,121.1,121.3,122.0,122.5,122.8,130.5,271.0,570-573,751,774.4,789.1,794.8,864,902,964.1,996.82, V02.6X, V42.7, V05.3	A06.4,A51.45,A52.74,B15-B19,B26.81,B58.1,B66,B67,D89,E74,K70-K77,Q44,R16,R94.5,T86,Z22.5X,Z94.4
Biliary stone	574	K80
CCI score		
Myocardial infarct	410	I21-22

Congestive heart failure	428.0, 428.1, 428.9	I50
Cerebrovascular disease	430–438	I60- I63, I65-I69, G45-G46
Connective tissue disease	710,714,725	M05, M06, M32, M33.20, M33.29, M34, M35.3
Peripheral vascular disease	250.7, 440.2-3,440.8-9, 443,444.22,444.8,447.8-9	E08.51-52, E08.59, E09.51-52, E09.59, E10.51-52, E10.59, E11.51-52, E11.59, E13.51-52, E13.59, I70.2-I70.9, I73, I74.2-I74.9, I75.011-I75.029, I75.89, I77.3, I77.89, I77.9, I79.1, I79.8
Dementia	290	F03.90, F05, F01.50,F01.51
COPD	490–492, 496	J40-J44, J47
Ulcer disease	531.XX, 532.XX, 533.XX ,534.XX	K25.X,K26.X,K27.X,K28.X,K31.82,K56.60
Mild liver disease	571.2,571.4X,571.5,571.6	K70.2,K70.3X, K73.X,K74.X,K74.60,K74.69,K75.4
Hemiplegia	342	G81
Moderate-to-severe renal disease	582.0,582.1,582.2,582.4,582.81,582.89,582.9,583.0, 583.1,583.2,583.4,583.6,583.7,583.9,588.1,588.8, 588.9, 583.81,583.89,585,586,588	E10.21, E11.21, N03.0, N03.1, N03.2, N03.3, N03.4, N03.5 N03.6, N03.7, N03.8, N03.9, N05.0, N05.1, N05.2, N05.3, N05.4, N05.5, N05.6, N05.7, N05.8, N05.9, N06.0, N06.1, N06.2, N06.3, N06.4, N06.5, N06.6, N06.7, N06.8, N06.9, N07.0, N07.1, N07.2, N07.3, N07.4, N07.5, N07.6, N07.7, N07.8, N07.9, N08, N14.0, N14.1, N14.2, N14.3, N14.4, N15.0, N15.8, N15.9, N16, N17.1, N17.2, N18.4, N18.5, N18.6, N18.9, N19, N25.0, N25.1, N25.81, N25.89, N25.9

Diabetes-with-end-organ-damage	250.4,250.5,250.6	E08.311, E08.319, E08.321, E08.329, E08.331, E08.339, E08.341, E08.349, E08.351, E08.359, E08.36, E08.39, E09.311, E09.319, E09.321, E09.329, E09.331, E09.339, E09.341, E09.349, E09.351, E09.359, E09.36, E09.39, E10.21, E10.22, E10.29, E10.311, E10.319, E10.36, E10.39, E10.40, E10.41, E10.44, E10.49, E10.610, E10.65, E11.21, E11.22, E11.29, E11.311, E11.319, E11.321, E11.329, E11.331, E11.339, E11.341, E11.349, E11.351, E11.359, E11.36, E11.39, E11.40, E11.41, E11.42, E11.43, E11.44, E11.49, E11.65, E13.21, E13.22, E13.29, E13.311, E13.319, E13.321, E13.329, E13.331, E13.339, E13.341, E13.349, E13.351, E13.359, E13.36, E13.39, E13.42, E13.43, E13.44, E13.49
Malignancy	140-149,150-159,160-165,170,174,175,176,179,180-189,190-195,200-201,203-208	C00-C96,D45, D47.Z9, E31.22, Z51.12
Moderate or severe liver disease	456.0,456.1,456.20,572.X,456.21,I85.XX,	K72.10,K72.11,K72.90,K72.91,K766,K767,K768.1
Metastatic solid tumors	196.X,197.X,198.X, 198.8X,199.X	C45.9, C77.X, C78, C79, C7A.00, C7A.094, C7A.095, C7A.096, C7A.1, C7A.8, C7B, C80.0, C80.1, D3A.8, J91.0, R18.0, Z51.12
AIDS	0.42,0.43,0.44	B20
Clinical outcomes		

Death	Linking to database of National Register of Deaths	
MACEs	410,426-427,428,430-438	I121-I122, I44-I49, I50, I60-I63, I65-I69
Ischemic stroke	433-435	I63, I65-I66, G45-G46

Abbreviation:

ICD, international classification of disease; COPD, Chronic obstructive pulmonary disease; CCI, Charlson Comorbidity Index; AIDS, Acquired immune deficiency syndrome; MACEs, major adverse cardiac events
